# Monomorphic T-cell post-transplant lymphoproliferative disorder with features of HHV8-negative primary effusion lymphoma: an autopsy case and review of the literature

**DOI:** 10.1007/s00795-024-00388-x

**Published:** 2024-05-23

**Authors:** Naoki Hosaka, Mitsuharu Hashimura, Atsuko Mugitani, Masanari Hamaguchi, Yuki Kubo, Shin-ichi Nakatsuka

**Affiliations:** 1 Department of Pathology, Fuchu Hospital, 1-10-7, Hiko-cho, Izumi, Osaka 594-0076 Japan; 2 Department of Hematology, Fuchu Hospital, 1-10-7, Hiko-cho, Izumi, Osaka 594-0076 Japan; 3 Department of Respiratory Medicine, Yao Tokushukai General Hospital, 1-17, Wakakusa-cho, Yao, Osaka 583-0011 Japan; 4 Department of Pathology, Yao Tokushukai General Hospital, 1-17, Wakakusa-cho, Yao, Osaka 583-0011 Japan; 5 Department of Hygiene and Public Health, Kansai Medical University, 2-5-1, Shin-machi, Hirakata, Osaka 573-1010 Japan

**Keywords:** Effusion lymphoma, HHV8 negative, T-cell type, Autopsy, Renal transplantation

## Abstract

A 67-year-old man underwent renal transplantation in his twenties. He developed refractory pleural effusion, with many large lymphocytes with severe atypia and mitosis in the effusion, indicating malignant lymphoma. He finally died of respiratory failure. An autopsy revealed atypical lymphocytes positive for CD3, CD4, and CD30 and negative for CD8, CD20, PAX5, human herpesvirus (HHV) 8, and Epstein–Barr virus-encoded small RNAs by immunohistochemistry and in situ hybridization. Atypical lymphocytes also had T-cell receptor gene rearrangements Jβ2, Jγ2, and Jδ1 and chromosomal aberrations der(8)t(1;8)(q21;p21), add(13)(q12), add(14)(q32), and add(16)(q12-13). A few atypical lymphocytes were present at other sites. We finally diagnosed this case as monomorphic T-cell post-transplant lymphoproliferative disorder with features of HHV8-negative primary effusion lymphoma. A literature review only identified six cases (four HHV8-negative, two HHV8-positive) of effusion lymphoma of T-cell type, including the present case. Interestingly, about half of HHV8-negative and HHV8-positive cases had a history of renal transplantation in their twenties. All cases showed tumor CD30 expression, whereas CD4 and CD8 expressions were inconsistent. These findings indicated that this lymphoma may be associated with post-transplant lymphoproliferative disorder by renal transplantation at a young age, although further cases need to be analyzed.

## Introduction

Primary effusion lymphoma (PEL) is a rare type of large B-cell lymphoma that occurs exclusively in body-cavity fluids, such as pleural effusion, ascites, and pericardial fluid [1]. PEL is highly associated with human herpesvirus (HHV) 8 infection and most often occurs during states of immunodeficiency caused by human immune deficiency virus (HIV) and/or Epstein–Barr virus (EBV) infection [2]. HHV8-negative cases of lymphoma have also been reported [3, 4], which are called effusion-based lymphoma (EBL), because PEL is primarily defined by the presence of HHV8 infection. In contrast, a recent report showed that most cases of effusion lymphoma (EL) occurred in patients with neither HHV8 nor HIV infection [5], although this study was limited to Japan. In addition, the tendency to fluid effusion due to conditions, such as renal failure, cirrhosis, and heart failure, is recognized as a background disease in patients with HHV8-negative EBL, with a favorable prognosis [4, 5]. With regard to lymphocyte type, only five cases of T-cell-type EL have been reported, irrespective of HHV8 infection [6–10], and these were not well classified. Furthermore, the pathogenesis of T-cell-type EL is poorly understood.

We experienced an autopsy case of monomorphic T-cell post-transplant lymphoproliferative disorder (PTLD) with features of HHV8-negative PEL in a 67-year-old man, who had a history of renal transplantation (RT) in his 20s. Interestingly, three out of six cases of T-cell-type EL, including the current case, had similar histories of RT, and HHV8 infection was not relevant. Here, we discuss T-cell-type EL related to PTLD [11].

## Clinical summary

A 67-year-old man underwent renal transplantation at 26 years of age. He also had percutaneous coronary interventions at 51 and 62 years of age. He had been on dialysis from 66 years of age and underwent left lower limb amputation because of arteriosclerosis obliterans at 67 years of age. He developed refractory pleural effusion 2 months prior to the current presentation, and the immunosuppressant drug azathioprine was discontinued after the start of symptoms because of the detection of methicillin-resistant *Staphylococcus aureus* (MRSA) in his foot lesions. He was diagnosed with T-cell lymphoma at another hospital because of atypical lymphocytes in the pleural effusion based on immunohistochemical, flow cytometric, and genetic analyses (data below). He was referred to our hospital, but dyspnea appeared and progressed rapidly. He died after experiencing respiratory symptoms for 2 days, and an autopsy was performed in our hospital.

The laboratory data from blood taken at the time of admission were as follows: white blood cells: 7000/mL (84% segment, 7% monocytes, 9% lymphocytes, 0% eosinophils, and 0% basophils); red blood cells: 292 × 10^4^/mL; hemoglobin: 9.1 g/dL; hematocrit: 30.2%; platelets: 22.3 × 10^4^/mL; reticulocytes: 24.4%; aspartate aminotransferase: 24 U/L; alanine aminotransferase: 19 U/L; alkaline phosphatase: 295 U/L; γ-glutamyl transpeptidase: 30 U/L; total bilirubin: 0.39 ng/dL; Na: 138 mEq/L; K: 6.1 mEq/L; Cl: 107 mEq/L; lactate dehydrogenase: 350 U/L; total protein: 6.4 g/dL; albumin: 2.8 g/dL; creatine: 5.74 mg/dL; soluble interleukin-2 receptor: 35,901 U/mL; C-reactive protein: 6.1 mg/dL; hepatitis B surface antigen (−), hepatitis C antibody (−), and HIV antibody(−). Human T-cell lymphotropic virus type 1 (HTLV-1) antibody was not examined.

The laboratory data from the pleural effusion were as follows: number of cells: 468/mL (78.5% mononuclear cells and 80.9% atypical cells); lactate dehydrogenase: 2022 U/L; adenosine deaminase: 52.4 U/L; and cytokeratin 19 fragment: 4.29 ng/mL. Flow cytometric analysis showed > 90% positivity for the pan-T-cell markers CD2, CD3, and CD5, and < 3% positivity for the pan-B-cell markers CD19 and CD20. CD4^+^ cells comprised 49.4% and CD8^+^ cells comprised 41.6% of total T cells.

## Pathological findings

No obvious lymphadenopathy or tumor mass was seen in any tissues or organs. Severe pleural effusion was observed in the left (1150 mL, bloody) and right (100 mL, bloody), although there was little ascites and pericardial fluid.

**Fig. 1:**
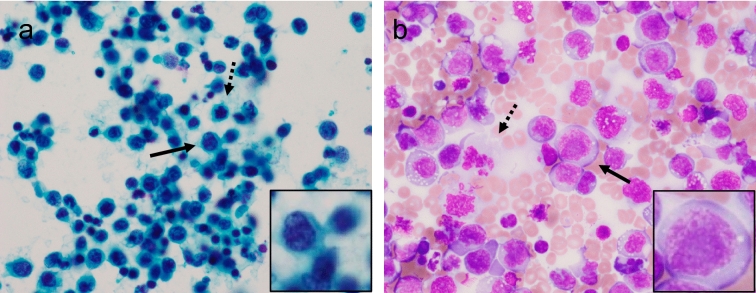
Cytological findings in the pleural effusion assessed by Papanicolaou’s and Giemsa staining. Papanicolaou’s (**a**) and Giemsa (**b**) staining are shown (× 400). Numerous large lymphocytes with atypia (arrow) and frequent mitotic cells were observed (dotted arrow). Inset: high magnification of area indicated by arrow.

**Fig. 2:**
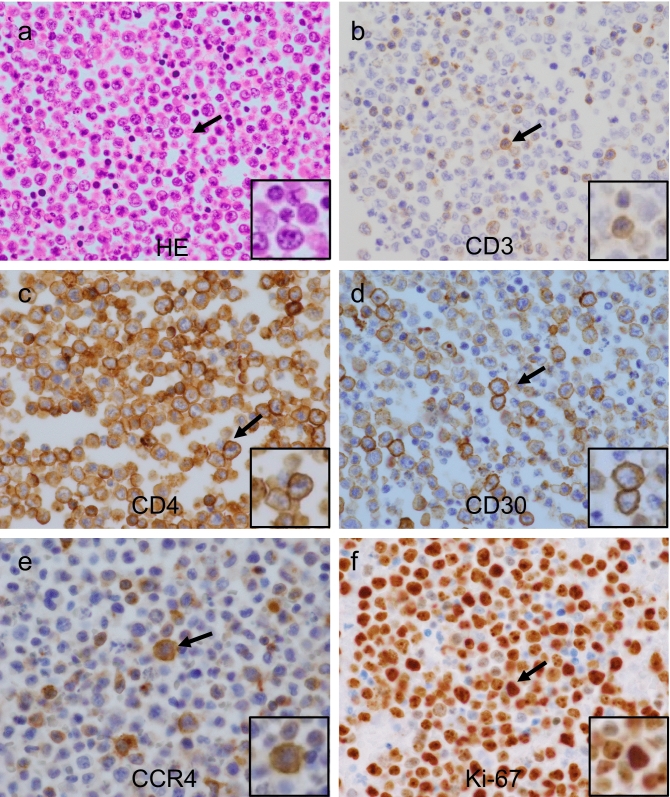
Histological and immunohistochemical analyses of lymphocytes from the pleural effusion. Hematoxylin and eosin (**a**) and immunohistochemical (**b** CD3; **c** CD4; **d** CD30; **e** CCR4; **f** Ki-67) staining shown (× 400). Cells with positive immunohistochemical staining indicated by arrows. Inset: high magnification of area indicated by arrow.

**Fig. 3:**
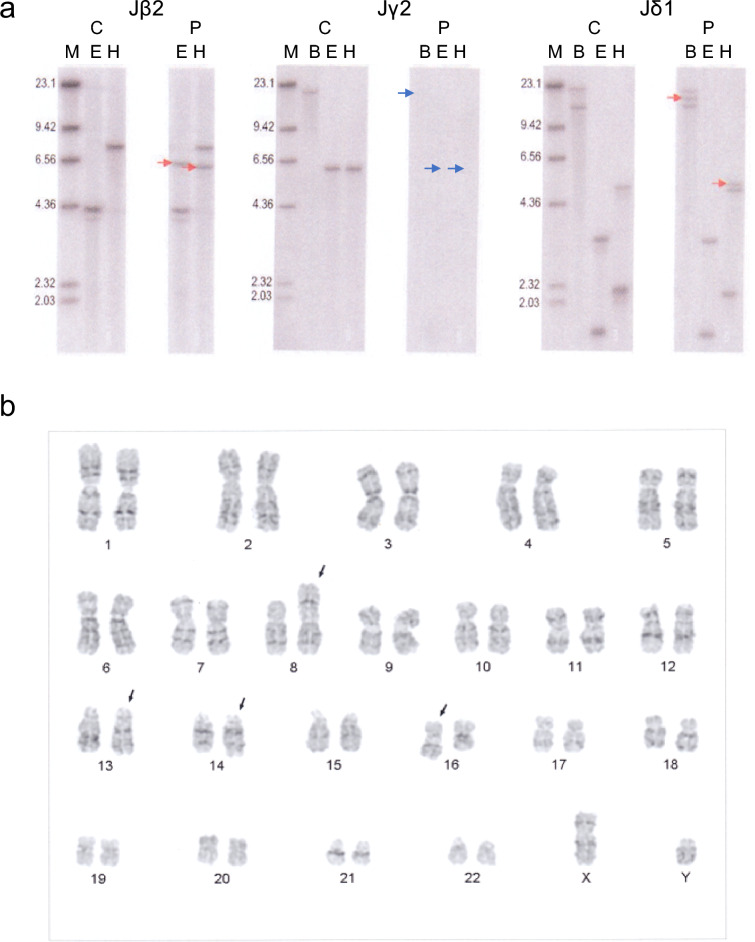
TCR rearrangements detected by Southern blotting and cytogenetic analysis by Giemsa C-banded karyotyping in lymphocytes from the pleural effusion. Control and patient samples were analyzed by Southern blotting (**a**). Rearranged bands are shown (red arrows). Relevant genes were deleted by rearrangement (blue arrow). C: control sample; P: patient sample; M: marker; B: *Bam*HI: E: *Eco*RI; H: *Hin*dIII. Cytogenetic analysis performed by Giemsa C-banded karyotyping (**b**). The following chromosomal aberrations were observed: 46XY, der(8)t(1;8)(q21;p21), add(13)(q12), add(14)(q32), and add(16)(q12-13) (black arrows).

Numerous atypical large lymphocytes were present in cytological specimens of the effusion, as assessed by Papanicolaou’s and Giemsa staining (Fig. 1a, b). Mitotic cells were also observed frequently. Numerous atypical large lymphocytes were also present in cell-block specimens (Fig. 2a). Immunohistochemical analysis indicated that these lymphocytes expressed CD3, CD4, CD30, and C-C chemokine receptor type 4 (CCR4) and the rate of Ki-67 expression was approximately 80% (Fig. 2b–f). Additionally, LCA, CD7, CD25, CD38, Bcl-2, and MUM1 were positive, whereas CD8, CD10, CD15, CD20, CD56, CD79a, CD138, PAX5, ALK1, CMV, EMA, FOXP3, HHV8, TIA-1, and EBV-encoded small RNAs were negative (Table 1). Southern blot analysis of DNA prepared from the effusion showed some clonal rearrangements in the T-cell receptor (TCR) Jβ2, Jγ2, and Jδ1 genes (Fig. 3a), but not in the Cβ1 and Jβ1 genes (data not shown). Cytogenetic analysis of the effusion by Giemsa C-banded karyotyping showed the following chromosomal aberrations: 46XY, der(8)t(1;8)(q21;p21), add(13)(q12), add(14)(q32), and add(16)(q12-13) (Fig. 3b).

**Table 1:** Summary of immunohistochemistry analysis

Result	Target
Positive	LCA, CD3, CD4, CD7, CD25, CD30, CD38, Bcl-2, CCR4, MUM1
Negative	CD8, CD10, CD15, CD20, CD56, CD79a, CD138, PAX5, ALK1, CMV, EMA, FOXP3, HHV8, TIA-1, EBER^a^

*LCA* leukocyte common antigen, *Bcl-2* B-cell lymphoma 2, *CCR4* C-C chemokine receptor type 4, *MUM1* multiple myeloma oncogene 1, *PAX5* paired box gene 5, *ALK1* anaplastic lymphoma kinase 1, *CMV* cytomegalovirus, *EMA* epithelial membrane antigen, *FOXP3* forkhead box P3, *TIA-1* T-cell-restricted intracellular antigen-1, *EBER* Epstein–Barr virus-encoded small RNA

^a^Analyzed by in situ hybridization

No such atypical lymphocytes were observed in other examined organs, including the bone marrow, pleura, diaphragm, heart, lung, spleen, digestive tract, liver, gallbladder, kidney, adrenal gland, aorta, skin, brain, pituitary gland, and the related lymph nodes. The liver showed no cirrhosis. Given the patient’s history of RT, we finally diagnosed monomorphic T-cell PTLD with features of HHV8-negative PEL [11, 12].

## Discussion

The present study reports a rare case of monomorphic T-cell PTLD with features of HHV8-negative PEL identified after autopsy. Notably, the lymphoma was neither B-cell-type nor associated with HHV8, which account for most cases of EL. Interestingly, this patient underwent RT in his 20s, and several previous studies of T-cell-type EL showed a similar history of transplantation, regardless of HHV8 infection [6–10]. We consider that some cases of this type of EL may have a different mechanism and/or pathogenesis of PTLD from classical PEL.

To the best of our knowledge, only six cases of T-cell-type EL have been reported, including our case (Table 2). Four cases were HHV8-negative and two were HHV8-positive, indicating that HHV8 infection was not necessarily related to the occurrence of the lymphoma. The main region of the effusion was the abdomen and/or pleural cavity, but not the cardiac cavity. Notably, half of all cases had a history of RT in their twenties, irrespective of HHV8 infection.

**Table 2:** Summary of clinical and pathological analyses of T-cell-type EL

Case	Age (years)/sex	Effusion and clinical information	RT (age, years)	EBV	HIV	HTLV-1	Prognosis	References
HHV8(−)								
1	27/F	Ascites	+ (22)	–	–	ND	3 weeks due to postoperative complications	[6]
2	72/F	Pleural, ascites	–	–	–	–	5 months	[7]
3	81/M	Pleural	–	–	–	–	2 years due to lung cancer	[8]
4	67/M	Pleural	+ (27)	–	–	ND	2 months	Present case
HHV8(+)								
5	49/M	Ascites	+ (20)	–	–	ND	≥ 10 months after CHOP	[9]
6	73/M	Pleural, LC	–	–	–	ND	Unknown (hospice service)	[10]

Case	Morphology	Immunohistochemistry^a^	Cytogenetics	Gene rearrangement TCR : IgH	References
HHV8(−)					
1	Immunoblastic/anaplastic	CD3, CD8, CD30	ND	(Oligoclonal)^b^ : None^b^	[6]
2	Large	CD3, CD7, CD30, TCR	del(1) (p11p22), +i(7)(q10), t(11;14)(q23;q11)	Cβ1^c^, Jγ^c^ : ND	[7]
3	Medium to large	CD3, CD4, CD7	ND	Cβ^c^ : None^c^	[8]
4	Large	CD3, CD4, CD7, CD30, CCR4	See text	Jβ2^c^, Jγ2^c^, Jδ1^c^ : ND	Present case
HHV8(+)					
5	Large/anaplastic	CD3, CD30, CD138, PAX5	ND	Γ^b^ : IgH^b^	[9]
6	Large	CD3, CD4, CD30	ND	Γ^b^ : ND	[10]

*CHOP* cyclophosphamide, doxorubicin (adriamycin), vincristine (oncovin), and prednisolone, *RT* renal transplantation, *EBV* Epstein–Barr virus, *HIV* human immunodeficiency virus, *HTLV-1* human T-lymphotropic virus, *TCR* T-cell receptor, *LC* liver cirrhosis, *ND* not determined

^a^CD7 not evaluated in cases 1, 5, and 6

^b^Polymerase chain reaction

^c^Southern blot analysis

No patients had EBV, HIV, or HTLV-1 infection, although these were not evaluated in all cases. Two patients had very short survival times of < 6 months, whereas one case survived for ≥ 2 years, while the survival was not reported for the other cases. Survival is difficult to assess due to the small number of cases, but it should be noted that survival was short, at least in some cases.

Pathological analysis indicated that all cases harbored immunoblastic/anaplastic or large tumor cells that expressed CD3 and CD30 by immunohistochemical analysis; however, expression levels of the T-cell subset markers CD4 and CD8 were inconsistent, and some cases did not express either. Cytogenetic analysis was carried out in two of the cases examined. All cases showed one or more critical TCR gene rearrangements in T-cell-type lymphoma; notably, however, this did not always reflect the phenotype of lymphoma, although it is unknown whether all TCR types were examined. Case 5 had TCR and Ig rearrangements with HHV8 infection. It was therefore difficult to determine if the infection was associated with T-cell -or B-cell-type neoplasms. These results suggest that most lymphomas have anaplastic or large tumor cells expressing CD30, with varied expression of the subset markers CD4 and CD8.

The significance of CCR4 expression in tumor cells was unclear in the current case. In this respect, we cannot exclude adult T-cell leukemia/lymphoma (ATLL), given that the presence of HTLV-1 was not undetermined as other cases in Table 2. In addition, some T-cell lymphomas are difficult to distinguish from ATLL, including PEL-like type [13, 14]. Care may be needed in handling these cases, although CD7-positive or FOXP3-negative phenotypes were not typical for ATLL in some cases in Tables 1 and 2, including cases in which CD7 was not evaluated.

The mechanism of HHV8-negative T-cell-type EL remains uncertain. The tendency to develop fluid retention due to renal failure, cirrhosis, and heart failure is thought to be associated with the development of primary HHV8-negative EBL [4, 5]. Additionally, the continuous use of immunosuppressive drugs may have a cumulative influence on several developing T-cell subsets in the juvenile thymus under renal dysfunction. This may lead to mutations in various T-cell genes. The present case also showed multiple rearrangements, including the TCR β, γ, and δ genes, although only one β-type lymphoma was apparent. Tumor cells may easily infiltrate into the pleural and/or abdominal cavity through the thoracic duct and chyle cistern via lymph ducts from the thymus.

In summary, we experienced a case of monomorphic T-cell PTLD with features of HHV8-negative PBL. A half of previous cases of HHV8-negative T-cell-type EL have been associated with PTLD, especially among patients with a history of RT at a young age. CD30 expression may lead to the administration of CD30 therapeutic antibodies [15]. These findings may offer new insights into the pathogenesis, diagnosis, and/or treatment of this type of lymphoma.

## Abbreviations

EL: Effusion lymphoma
PTLD: Post-transplant lymphoproliferative disorder
PEL: Primary effusion lymphoma
RT: Renal transplantation
ATLL: Adult T-cell leukemia/lymphoma
